# Exploratory Testing of Diatom Silica to Map the Role of Material Attributes on Cell Fate

**DOI:** 10.1038/s41598-017-13285-4

**Published:** 2017-10-26

**Authors:** Pamela J. Walsh, Susan A. Clarke, Matthew Julius, Phillip B. Messersmith

**Affiliations:** 1 School of Chemistry & Chemical Engineering, Queen’s University, Belfast, UK; 2 Biomedical Engineering Department, Northwestern University, Evanston, Illinois USA; 3 School of Nursing & Midwifery, Queen’s University, Belfast, UK; 4 Biological Sciences, St. Cloud State University, St. Cloiud, MN USA; 5 Departments of Bioengineering and Materials Science and Engineering, University of California, Berkeley, California USA

## Abstract

Porous silica is an attractive biomaterial in many applications, including drug-delivery systems, bone-graft fillers and medical devices. The issue with porous silica biomaterials is the rate at which they resorb and the significant role played by interfacial chemistry on the host response *in vivo*. This paper explores the potential of diatom-biosilica as a model tool to assist in the task of mapping and quantifying the role of surface topography and chemical cues on cell fate. Diatoms are unicellular microalgae whose cell walls are composed of, amorphous nanopatterned biosilica that cannot be replicated synthetically. Their unique nanotopography has the potential to improve understanding of interface reactions between materials and cells. This study used Cyclotella meneghiniana as a test subject to assess cytotoxicity and pro-inflammatory reactions to diatom-biosilica. The results suggest that diatom-biosilica is non-cytotoxic to J774.2 macrophage cells, and supports cell proliferation and growth. The addition of amine and thiol linkers have shown a significant effect on cytotoxicity, growth and cytokine response, thus warranting further investigation into the interfacial effects of small chemical modifications to substrate surfaces. The overall findings suggest diatom-biosilica offers a unique platform for in-depth investigation of the role played by nanotopography and chemistry in biomedical applications.

## Introduction

The use of implantable silica biomaterials is becoming more widely accepted by the scientific community and is gaining more clinical support with translation of these devices to the marketplace, e.g. commercialised bioglass^®^ 45S5^1,2^ and Si-substituted pastes^3^. Bioglass^®^ 45S5 (trade name PerioGlas^®^) developed by Larry Hench^1^ is one of the most successful silica-based products used clinically^4^. The use of porous silica as a drug-delivery system *in vivo* became popular in the mid 1990s following an influential study by Canham^5^, although silica based drug-delivery systems have yet to progress to a commercial market.

The body can tolerate and eliminate silicic acid^5^, and in some instances, e.g. bone repair, the release of silicon-containing species is thought to induce a therapeutic response^6,7^. An important challenge in this field is understanding the resorption profile of the silica substrates, which is largely controlled by surface chemistries^5^. The influence of material attributes on cellular behaviour and physiochemical properties has been widely reported^8,9^. Subtle changes, e.g. surface chemistry, can result in significantly different biological responses^10^ and many studies have shown well ordered nanotopographies, such as those produced by nanolithography^11^, can increase macrophage adhesion, cytoskeletal morphology and cytokine expression, while reducing oxygen species production. Other studies have reported inhibitory effects on macrophage activity (e.g. adhesion, viability and proliferation)^12^. While it is tempting to draw parallels between these studies, the mechanism of silicon ion release, the interplay of material attributes (e.g. biosilica spicules^13,14^ to synthetic nanoparticles^10^) and the variation in their chemical structures makes comparison difficult. In most studies, the release profile has not been sufficiently quantified to ascertain its therapeutic concentration, if indeed the biological response is a direct result of Si ion dissolution. Furthermore, although, these types of studies are useful when investigating the inter and intra-cell signalling pathways from the direct release of Si ions, they do not account for the interaction of the cells with the delivery system.

Diatom biosilica offers a unique opportunity to study the mechanism of Si ion release, from a particulate biosilica source that has the added advantage of ‘built-in’ nano-topographical functionality. Diatoms are unicellular algae that synthesise species-specific amorphous silica cell walls known as frustules^15,16^ that are identically replicated from generation to generation^17,18^. These diatom frustules are mineralised on an organic template, that is subsequently bound together with an organic matrix^18–20^. The frustules consist of two overlapping valves and span a range of sizes from 1 μm to 2 mm, depending on species^20^. It would be impossible to fabricate structures with such precision, uniformity and complexity synthetically. In addition, the fabrication of synthetic silica used in biomedical applications, e.g. mesoporous silica, requires toxic chemicals (in particular hydrofluoric acid)^21^ which result in particles with limited topography and high size variation.

There are currently estimated to be over 200,000 different diatom species, each with their own unique shape and morphology^15,18,22^. This offers a huge array of surface topographies, particle sizes and shapes, which could be used to help understand the role of silica in bone repair and the influence of material attributes on the cell response. A recent study by Cicco *et al*., tested the cell response of the diatom *Thalassiosira Weissflogii* using osteoblastic (Saos-2) and fibroblastic (NHDF) cell lines, and found no adverse cellular response to diatom frustules^23^. Cicco’s study provides a useful insight into the cellular biocompatibility of diatoms as an orthopedic implant or wound healing material, however, does not investigate the pro-inflammatory response, which is the most common cause of implant failure^24^.

We aimed to investigate cytotoxicity, cell viability, proliferation and cytokine responses of cells directly exposed to diatom silica frustules. For this study, *Cyclotella meneghiniana* was selected, a centric diatom with an average particle size of 20 µm^25^. The rationale was to minimise phagocytosis by macrophages, a process which is size and shape dependent^26^, and assess the cells’ interaction with the surface of the frustules. Functionalisation of the external surface of silicon based drug-delivery systems, and/or implantable materials, has been shown to be critical for biomedical applications to improve their versatility^21,27^. In this study, frustules were functionalised with amino and thiol end groups following isolation from their organic matrix.

## Results and Discussion

For this study, *C meneghiniana* was isolated from the Mississippi River, USA and cultured through several growth cycles. Samples were grown under controlled conditions in a closed photo bioreactor system using modified WC media (composition in Supplementary Table S1) specific for algal culture growth with a Na_2_SiO_3_.9H_2_O precursor in purified freshwater. The bulk composition of the diatom frustules was screened using Inductively Coupled Plasma optical emission Spectrometry (ICP-OES) for heavy metal contamination (Supplementary, Table S2). Si ions, as expected, were the most abundant ions, 383,178(±932) mg/kg, detected in the frustules, followed by Ca ions, which were detected at a concentration of 19,929(±297) mg/kg. Arsenic was present at 3.7(±1.2) mg/kg, however, can be controlled in the culture phase as it is metabolised by the algal cell from its surroundings. If this material is deemed suitable for implantation, the arsenic content could be reduced so as to be almost negligible. In diatoms, Cd is thought to act as a chelating agent to create a barrier against potentially toxic metal ions^28,29^. No Cd was detected in our samples, however, elevated levels 140.2(±43)mg/kg of Zn were detected which could have been substituted for Cd. The source of this Zn is unknown but owing to their similar chemical and physical properties, Zn can displace Cd and vice-versa from metallo-enzymes in the diatom frustules^28^ or may be present in frustules via other mechanisms^30^. Alternatively, if the acid digest was incomplete and residual organic material is present, this could account for the levels of Zn as it is associated with finger proteins in the cytoplasm of diatoms^31^. However, no other elements, such as Cl, Cu and S that are also associated with cytoplasm were detected, therefore this hypothesis is unlikely. In biomaterials, Zn in trace amounts is considered desirable as it has anti-microbial properties^32^, whereas Cd is considered toxic.

**Figure 1:**
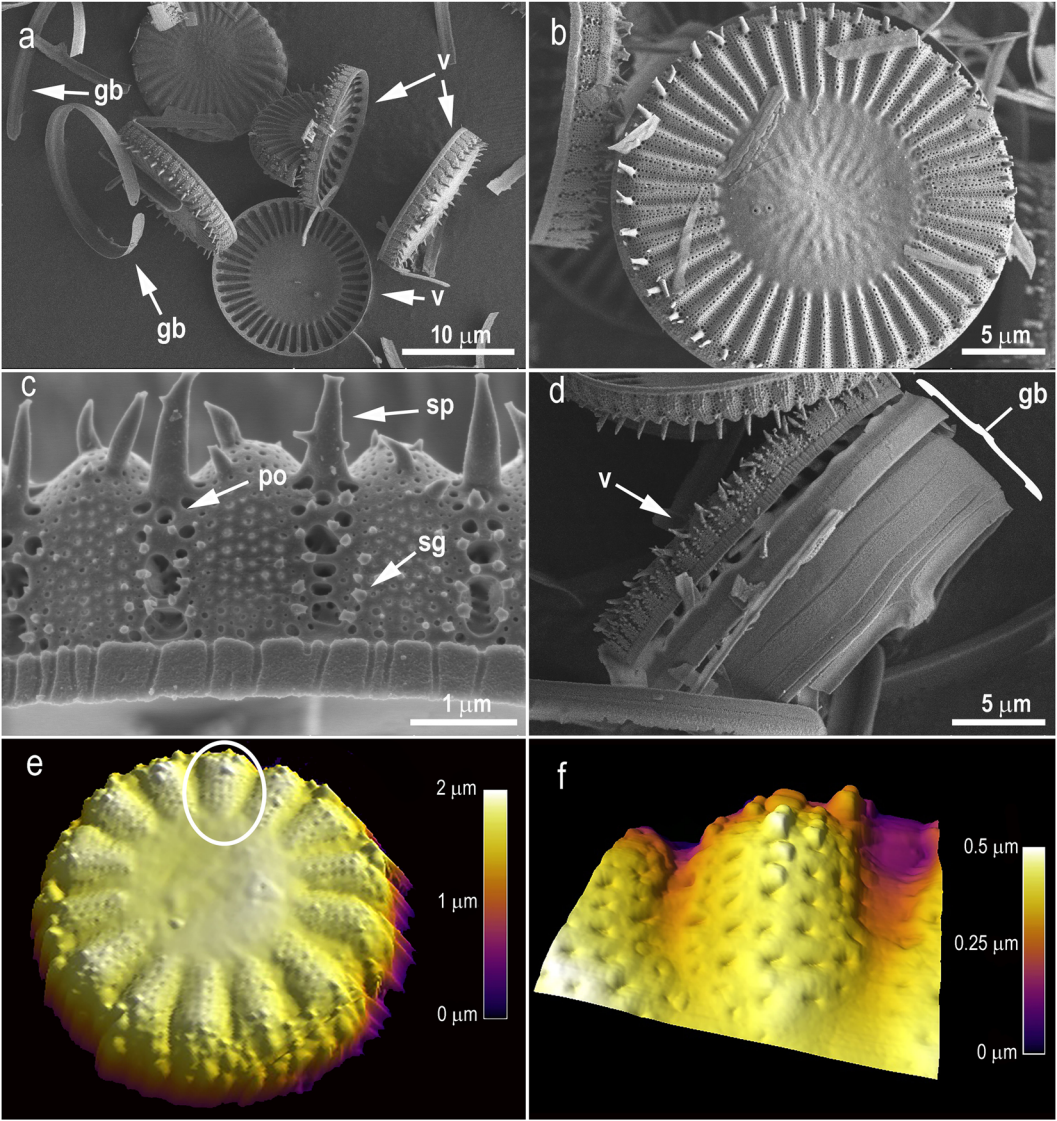
*Cyclotella meneghiniana* cell wall visualisation. Scanning electron micrographs (a–d): (**a**) Valves (v) and girdle bands (gb) from deconstructed diatom frustules. (**b**) External valve view complete frustule 22 μm. (**c**) Valve margin showing process openings (po), silica granules (sg), and spines (sp). (**d**) Upper valve (v) with girdle bands intact (gb). Atomic force micrographs (**e,f**) illustrating C. meneghiniana’s true 3D cell wall morphology. (**e**) Valve face of complete frustule 7 μm, circle indicates area panel d enlargement. (**f**) 1.5 μm ×1.5 μm scan of cell wall alveolous.

*C meneghiniana* a centric diatom (Fig. 1) was chosen as a model for this study due to availability of biomass. All diatom frustules are comprised of two overlapping biosilica valves linked by biosilica girdle bands (Fig. 1a and d). This structure is bound together by 62 (±2.12) % organic material (Supplementary Fig. S1). This organic material was removed (verified by TGA, Supplementary Fig. S1) using a nitric acid digest to expose the nanotopographical features of the diatom frustules and reduce cytotoxicity issues associated with it. However, attempts to isolate the valves from the girdle bands were unsuccessful therefore samples were tested as a crude mixture (Fig. 1a). The valves of the diatom also have a range of interesting topographical features, including sharp spicules (Fig. 1c) with porous radial striated surfaces towards the outer valves (Fig. 1b). Towards the centre of the diatom frustules is a smoother and uniseriate surface (Fig. 1b). Atomic force microscopy (AFM) analysis indicates closed-pore topography on the outer surface (Fig. 1e,f) that creates a nanopatterned, textured surface. Whereas the SEM image in Fig. 1c clearly shows both open and closed pores, with some interconnectivity between pores directly below the spines. The upper valve has an approximate depth of just under 2 μm as indicated by AFM (Fig. 1e), whereas the lower valve appears to be much deeper (Fig. 1d).

The morphological features on this diatom resulted in a low surface area, which was found to be ~33.67m^2^g^−1^ (n = 3) with a pore size distribution ranging from 1.6 to 30 nm (Supplementary Fig. S2). The valve size of *C meneghiniana* imaged in Fig. 1b is just over 20 μm, whereas the AFM image is taken from a frustule at the smaller end of the range, with a valve diameter of approximately 7 μm. As diatoms age, reductive cell division lowers the average size of individual valves in a population, and thus alters their frustule dimensions^33^. For this study, diatoms were harvested when the valves were approximately 20 μm in diameter, verified by SEM (Supplementary Fig. S3).

**Figure 2:**
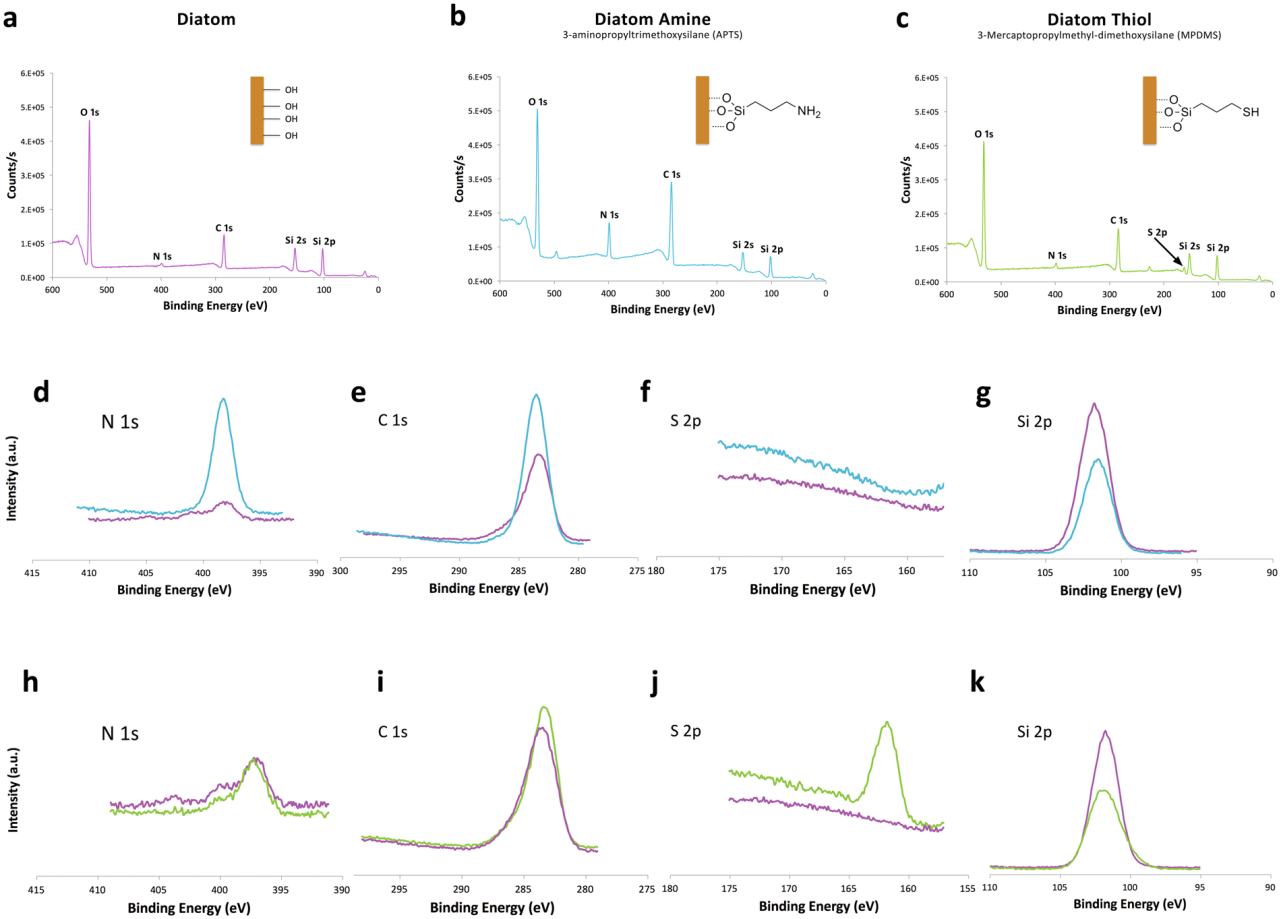
X-ray photoelectron spectroscopy survey scan (**a**) Diatom (**b**) Diatom Amine (linker APTMS), (**c**) Diatom Thiol (linkerMPDMS) at binding energy range of 0 to 600Ev (**d**) N 1s peak (**e**) C 1s peak (**f**) S 2p peak (**g**) Si 2p peak for diatom functionalised with amine compared to unfunctionalised, (**h**) N 1s peak (**i**) C 1s peak (**j**) S 2p peak (**k**) Si 2p peak for diatom unctionalised with thiol compared to unfunctionalised.

X-ray Photoelectron Spectroscopy (XPS) spectrum (Fig. 2) and relative atomic ratios (Table 1) confirm that functional groups: amine (–NH_2_) and thiol (–SH) were successfully grafted onto the surface of *C. meneghiniana*. The Si 2p peak for *C. meneghiniana* is consistent with the expected SiO_2_ 102.84 eV for silica and biosilica found in other diatom species, e.g. *Thalassiosira pseudonana* ^19^. For the unmodified *C. meneghiniana* frustules, an O/Si atomic ratio equal to 2 was observed, which is indicative for SiO_2_ materials. A similar atomic ratio was observed by Fowler *et al*., on the surface of diatomaceous earth^34^. Cicco *et al*.^23^ and Yu *et al*.^35^ however, observed higher ratios of 5 (*T. Weissflogii*) and 3 (diatomaceous earth) respectively. In Cicco *et al*., it is unclear if values quoted relate to atomic or mass ratios, making direct comparison difficult^23^. Their study also reported detecting sulphur (S/Si = 0.66) and chlorine (Cl/Si = 0.09) in unmodified frustules, which was not detected in this study (Tables 1 and 2, Supplementary 2 Fig. S1). Jonge *et al*., reported these elements to be associated with cytoplasmic pillars in diatoms as opposed to impurities in the inorganic silica frustule and suggest the presence of residual organic material in their samples^31^.

**Table 1:** X-ray photoelectron spectroscopy data for surface modification of *C. meneghiniana* grafted with functional groups.

	Diatom Substrate Used	Concentrations	Samples	C/Si	O/Si	S/Si	N/Si
This Study	*C. meneghiniana*	Atomic	**Diatom**	1.54	2.06	0	0.16
			**Diatom Amine**	4.17	2.65	0	0.97
			**Diatom Thiol**	2.88	2.26	0.41	0.22
Cicco^23^	*T. Weissflogii*	Undefined	**Diatom**	2.40	5.00	0.66	Not Reported
			**Diatom Amine**	16.00	4.80	0.73	Not Reported
			**Diatom Thiol**	3.39	2.60	0.40	Not Reported
Fowler^34^	Diatomaceous earth	Atomic	**Diatom Thiol**	5.32	2.01	0.64	0.11
Yang^11^	Diatomaceous earth	Atomic	**Diatom**	1.34	3.22	0	0
			**Diatom Amine**	1.55	2.62	0	0.17

**Table 2:** Carbon, Hydrogen, Nitrogen, Analysis for bulk characterisation of surface modification of *C. meneghiniana* grafted with functional groups (–SH and –NH_2_); n = 6.

Samples	%C	%H	%N	%S
Diatom	9.85 (±1.75)	2.09 (±0.34)	1.25 (±0.17)	<0.5
Diatom Amine	12.65 (±1.01)	2.30 (±0.11)	2.25 (±0.60)	<0.5
Diatom Thiol	11.09 (±0.7)	2.33 (±0.18)	<0.5	0.93(±0.25)

In this study, functionalised diatom frustules covered with silanol (Si-OH) groups showed an O/Si ratio increase to 2.26 (thiol) and 2.65 (amine). The O/Si ratio for thiol functionalised diatoms was found to be within the range (2 to 2.6) of those quoted in the literature^23,34^. For amine functionalised diatoms, the O/Si ratio was in good agreement with Yu *et al*., study^35^. An increase in C/Si ratio from 1.5 (unmodified) to 2.88 (thiol) and 4.17 (amine) was also observed. These results suggest the presence of silanol-linker on the surface of the diatom frustule, which is likely to cause an increase in C 1s (although carbon content is often overestimated in XPS due to contamination) and Si 2p chains. A N 1s peak 399.84 eV was observed in all three groups (Fig. 2d and h), however a more pronounced peak (Fig. 2d) was present in the group which was functionalised with amine (3-aminopropyltrimethoxysilane (APTMS)), (Fig. 2d). This would suggest the successful silanisation of an amine end group. A weaker S 2p peak was observed at 162.91 eV in samples that were functionalised with thiol (3-mercaptopropylmethyl-dimethoxysilane (MPDMS)), as shown in Fig. 2j. However, S 2p was not detected in the other two groups (Fig. 2f). These results suggest that the silanisation reaction has been successful for the respective linkers and this was confirmed by Carbon Hydrogen Nitrogen Sulphur (CHNS) analysis data (Table 2), which showed similar trends. Diatom frustules functionalised with the amine showed elevated levels of nitrogen (2.25 (±0.60) %), whereas those functionalised with thiol showed elevated levels of sulphur (0.93 (±0.25) %), thus indicating that the silanisation reaction has been successful.

**Figure 3:**
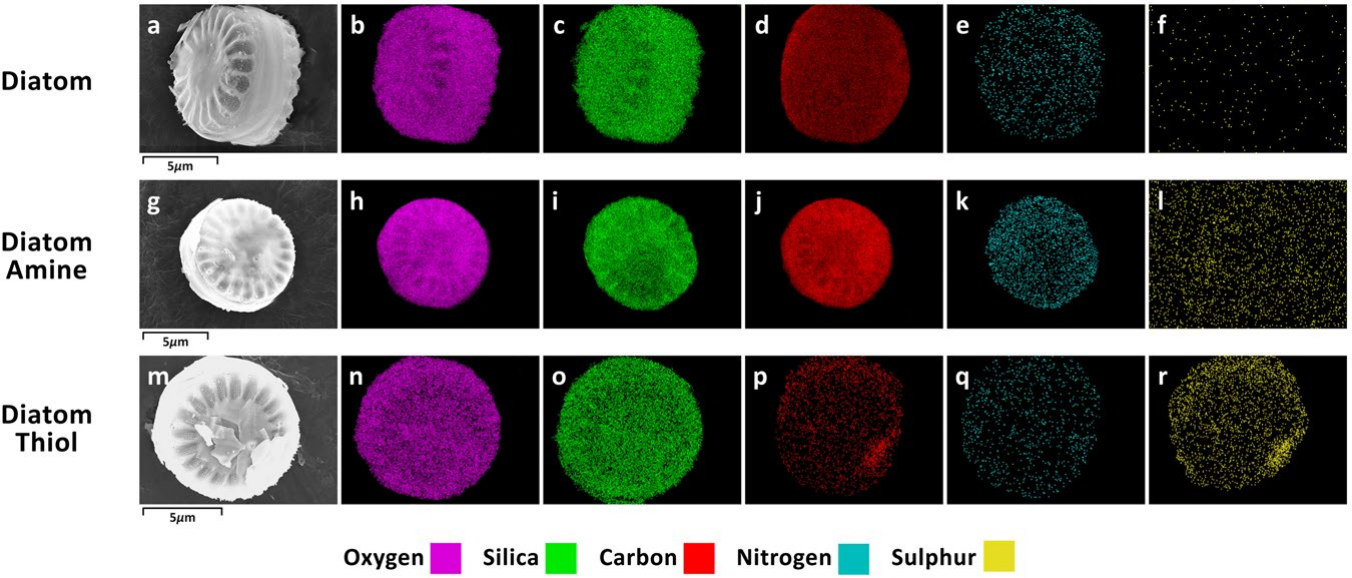
Scanning electron micrographs of C. meneghiniana (**a**) Diatom (**g**) Diatom Amine (linker APTMS), (**m**) Diatom Thiol (linker MPDMS). Energy-dispersive X-ray spectroscopy (EDS) elemental maps of diatom (**b**,**h**,**n**) oxygen signal, (**c**,**i**,**o**) silica signal, (**d**,**j**,**p**) carbon signal, (**e**,**k**,**q**) nitrogen signal, (**f**,**f**,**r**) sulphur signal. The scale bar in the SEM images (**a**,**g** and **m**) is the same scale for each image in the panel.

The specific surface area showed a threefold reduction in both surface modified groups (Supplementary 2 Fig. S2a) and a reduction in the average pore diameter from approximately 17nm to <10nm (Supplementary 2 Fig. S2b) confirming the presence of a surface coating. EDS elemental mapping (Fig. 3) for groups functionalised with APTS and MPDMS reveal a homogenous distribution of nitrogen (Fig. 3e and q) over the surface of the diatom frustule, and slight clumping of sulphur towards one corner of the outer surface (Fig. 3r) for the respective linkers. The signal for oxygen (Fig. 3d, j and p) and silica (Fig. 3c, i and o) was observed to be much stronger than the grafted elements sulphur (Fig. 3f, l and r) and nitrogen (Fig. 3d, j and p), indicating that a higher concentration of these elements was present. Other studies provide no indication of whether or not they achieved homogeneity in terms of the surface distribution of the linker on the diatoms^23,34,35^.

A mouse BALB/C monocyte macrophage cell line, J774.2 were selected to model physiological scavengers of foreign particles and to evaluate cellular response to unmodified and functionalised diatoms. This cell line has been extensively used to assess biological response to particles *in vitro* according to the American Standard Test Methods (ASTM) standard test 1903–98^36^. In order to assess the osteogenic capacity of diatom frustules, further cellular studies were carried out using primary human bone marrow stromal cells (hBMSC). These studies assessed cellular response of both J774.2 and hBMSC’s to different types of diatom frustules and conditioned media obtained from immersing diatoms frustules in culture media.

Prior to cellular studies the pH was found to fluctuate slightly over 3 days when diatom particles were suspended in RPMI culture media (without phenol red indicator) in a cell free environment (data not shown). For all cell studies, the diatom frustules were soaked in culture media for 24 h prior to cell seeding and the pH of the diatom suspension was adjusted to ~7.4 (with concentrated HCl) directly before use.

**Figure 4:**
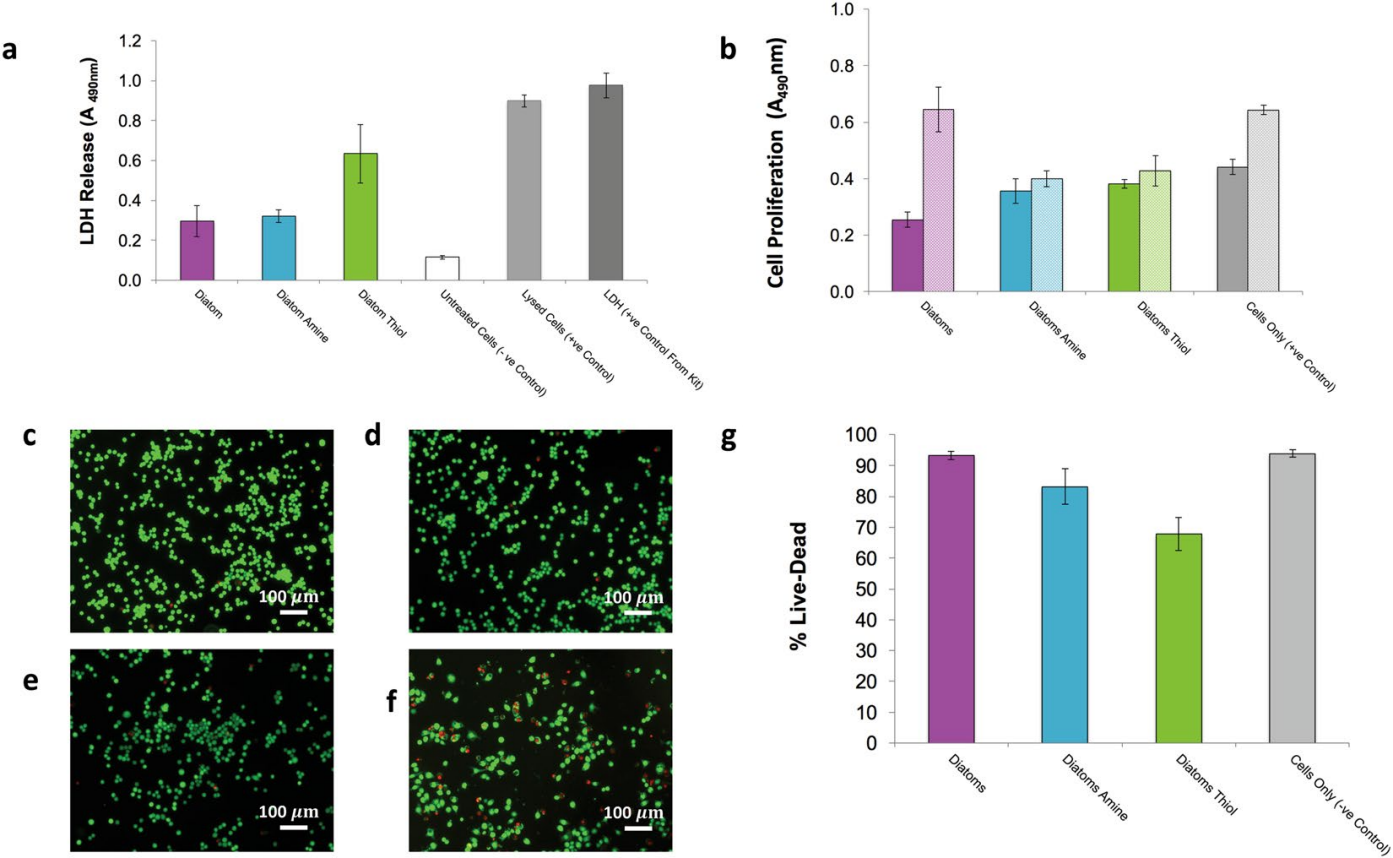
Biological Response of J774.2 macrophages grown on diatom frustules (**a**) LDH release measuring cytotoxicity after 24 hrs, (**b**) cell proliferation, solid bars represent 24 h and hatched bars represent 72 h in culture, Error bars indicate ±1 SD, N = 3 for **a** and **b**. (**c** to **f**) fluorescence microscopy images of live/dead stained J774 cells (green = live; red = dead) after 24 hrs in culture, (**c**) cells (**d**) Diatom (**e**) Diatom Amine (**f**) Diatom Thiol (**g**) quantitative J774 cell viability determined from image analysis. Error bars indicate ±1 SD. N = 6/image × N 3 replicate wells.

**Figure 5:**
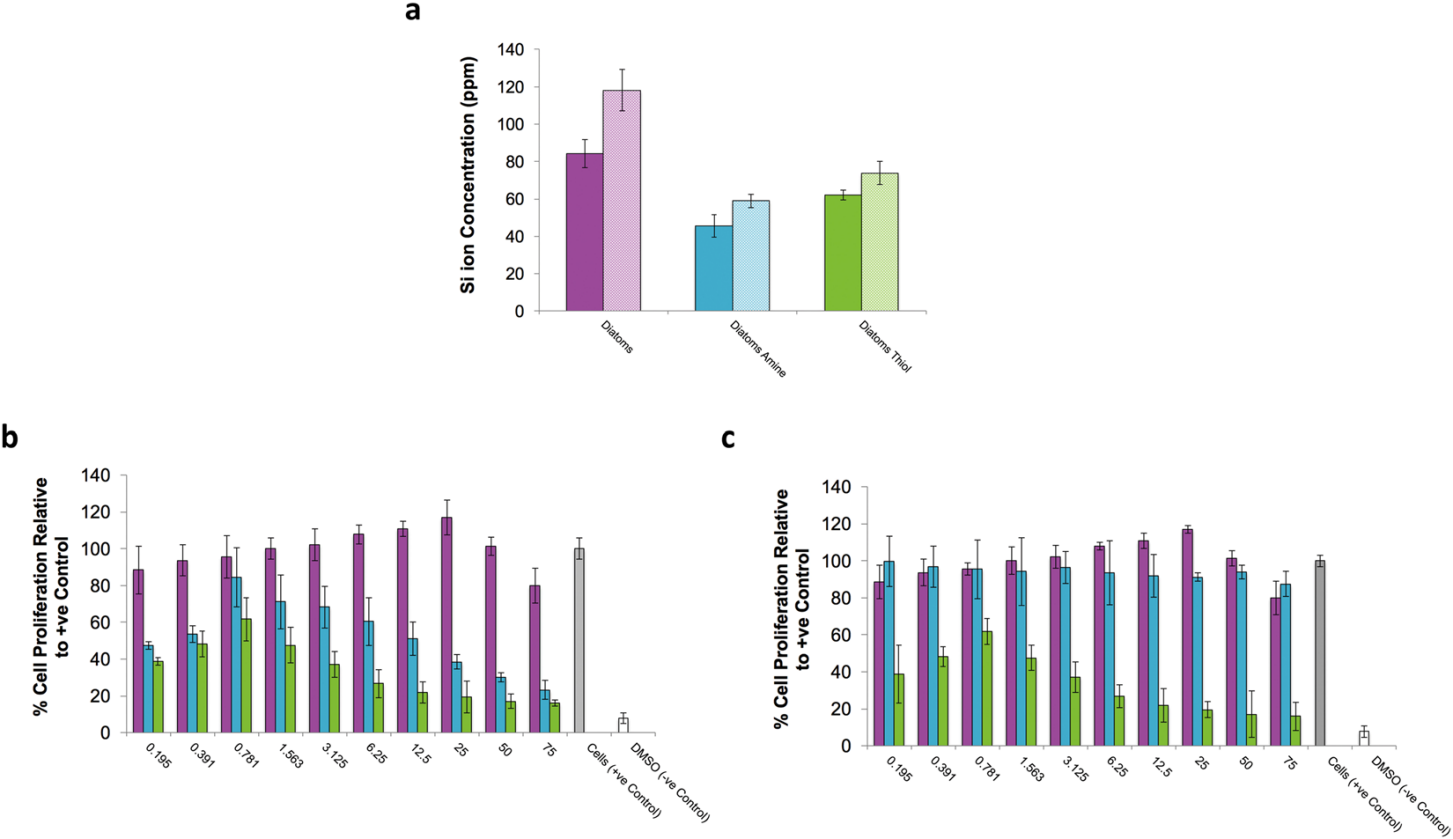
(**a**) Silica ion release profile of C. meneghiniana determined by Inductively coupled plasma mass spectrometry (ICP-OES), solid bars represent 24 h and hatched bars represent 72 h in culture. Error bars indicate ±1 SD, N = 3. Dose response profile of J774.2 macrophages (**b**) diatom frustules (C. meneghiniana) in culture for 24 h, (**c**) Conditioned media (C. meneghiniana) in culture for 24 h.

Cytotoxicity, cell viability, cell proliferation and cytokine responses were measured using J774.2 cell lines as shown in Figs 4 and 5. To assess cytotoxicity, lactate dehydrogenase (LDH) release was used to measure the damage to cell membrane. This type of cell death is known as necrosis and can occur in a matter of seconds^27^. The LDH released into the supernatant (Fig. 4a) was measured 24 hours after the J774.2 cells were seeded directly onto the frustules and was found to be within the range of the positive and negative controls for all groups. ANOVA revealed that there was a significant treatment effect (p = 8.206 × 10^−24^) with LDH release in the diatom and diatom amine groups significantly higher than untreated cells, and LDH release in diatom thiol group significantly higher than diatom and diatom amine. LDH release was six-fold higher in Diatom Thiol compared to Diatom Amine.

These results were confirmed by a cell viability test which was measured after 24 hours using Live/Dead staining (Fig. 4c–f) and quantified using image analysis to assess the total percentage of viable cells (Fig. 4g). Healthy cells uptake calcein AM which becomes hydrolysed and fluoresces green when imaged under a laser scanning confocal microscope (LSCM). Red fluorescent ethidium homodimer tags only enter cells with damaged cytoplasmic membranes, binding to DNA. The results show all groups support cell viability, however, slightly more red cells were visible in diatom frustules functionalised with thiol, shown in Fig. 4f. When quantified, it was found that cell viability for all groups was >90% with the exception of Diatom Thiol where the cell viability fell to 68.75(±4.59) % (Fig. 4g). Statistical analysis by ANOVA confirmed a treatment effect (p = 1.159 × 10^−21^) and post hoc testing showed that Cells Only and Diatom treatment groups were not significantly different from one another. Diatom Amine treatment group had a significantly lower cell viability than Cells Only and Diatoms Thiol treatment group had significantly lower cell viability than all other treatment groups.

Cell proliferation was determined using an MTS assay. In this experiment, there was no media change between 24 and 72h. The results (Fig. 4b) show that diatom frustules regardless of surface treatment, clearly affect cellular proliferation when compared to cell only control groups. After 24h, all diatom frustule groups appeared to have a significant reduction in proliferation (ANOVA p = 4.826 × 10^−12^). However, little difference was observed between the two groups of functionalised frustules, with a proliferation rate in both groups that was statistically significantly lower than the cell only control. Surprisingly, the Diatoms treatment group had a significantly lower absorbance than all other treatment groups at 24 h yet after 72 h, appeared to show a significant increase in proliferation compared to the two groups of diatoms that were functionalised, thus suggesting that something was having a stimulatory effect on the cells. This was confirmed by statistical analysis; there was no significant difference between Cells Only and Diatoms treatment groups at 72 h. Cell proliferation at 72 h in Diatoms Amine and Diatoms Thiol treatment groups, although significantly higher than at 24h (p = 0.002 and p = 0.011), was still significantly suppressed, in statistical terms, compared to Cells Only and Diatoms treatment groups.

The release of silica ions into the media was determined using inductively coupled plasma mass spectrometry (ICP-OES), as shown in Fig. 5a. The results showed higher silica ion release in diatoms without functionalisation compared to the two functionalised groups after both time points. After 24 h, silica ion release in the Diatom group was found to be 80.15(±7.42) ppm compared to 45.75(±5.98) and 62.07(±2.75) ppm released from Diatom Amine and Diatom Thiol respectively. After 72 h, the silica ion release rate increased by 28%, 22% and 15% in the Diatom, Diatom Amine and Diatom Thiol groups respectively. A t-Test found that silica ion release was significantly higher at 72 h compared to 24 h for individual treatments, matching the trend in cell proliferation (Diatoms p = 0.0001, Diatom Amine p = 0.0009 and Diatom Thiol p = 0.0016). Indeed, the trend observed in silica ion release was similar to that observed in relation to cell proliferation (Fig. 4b) with the highest levels of both in the Diatom group without functionalisation. This might suggest that the silica is having a stimulatory effect. The addition of linkers, clearly create a physical barrier that limits hydrolysis of siloxane bonds reducing silica ion dissolution. Surface functionalisation may also reduce the hydrophobicity of the surface of the diatom frustules. Several studies have reported that hydrophilic surfaces cause a reduction in the absorption of serum proteins, and therefore cell binding, resulting in a reduction in cell proliferation^12,37^.

To quantity this further, dose response analyses on both silica frustules and conditioned media (the same weight of silica was added to media for the same time period without cells, this media was then centrifuged, filtered and the solid mass – frustules- disregarded) were carried out over a 24h period to assess cell proliferation (Fig. 5b and c). The inhibitory effect that was observed in both functionalised groups when cultured with diatoms directly (Fig. 4a) was no longer observed for Diatom Amine in conditioned media. However, the inhibitory effect of Diatom Thiol on proliferation was maintained in both direct contact and conditioned media. These results follow the opposite trend to Cicco *et al*., who reported a better cellular response to diatoms functionalised with a thiol end group^23^. However, it is noteworthy that the thiol linkers used are slightly different in both studies. Cicco *et al*., have selected 3-mercaptopropyl-trimethoxysilane, whereas this study used 3-mercaptopropyl-methyldimethoxysilane. The difference between these two linkers is that 3-mercaptopropyl-trimethoxysilane has an oxygen, which in terms of functional difference, means that this linker has three sites as opposed to two sites for cross-linking or binding to a surface. Before conjecturing about the role of the oxygen in terms of cell response, it is also worth noting that there are differences in both the nanotopography of the diatom substrates used and the surface modification achieved in both studies.

Lin *et al*., also compares the cellular response of both amine and thiol linkers, and reported similar findings to our study^37^. Lin’s study concludes that amine end groups from the 3-aminopropyltrimethoxysilane linker promote better cell proliferation and growth owing to the close contact between the cells and the positively charged amino groups (−NH_3_^+^) on the substrate surface. The thiol linker used in their study, is the same linker that was used by Cicco *et al*.^22^ Neither of these two studies take into consideration the influence of the by-products from the substrate that is leached into the tissue culture media, and their influence on cellular response. Our results clearly show that the byproducts also influence cell proliferation. No differences were found in terms of the concentration of silica ion release from Diatom Amine compared to Diatom Thiol, however, in both the conditioned media experiment, and cell seeding directly onto diatom frustules, cell proliferation was significantly reduced in the Diatom Thiol group. Although, other factors such as surface charge, pH, change in nanotopography, impurities and/or release of organic moieties from the linkers are also likely to influence the cellular response on cells seeded directly onto solid substrates, the results from our conditioned media experiment suggest that some small molecule (<2 μm filter used) is leaching into the media and is in turn causing an inhibitory response in the Diatom Thiol group. The 3-mercaptopropyl-trimethoxysilane linker used by Cicco *et al*.^23^ has three methoxy groups that form 3 Si-O bonds to silica, creating three connections in three directions, this in theory should result in a stronger graft network. The linker used in this study, 3-mercaptopropyl-methyldimethoxysilane, has one Si-O bond less, therefore, has a weaker bond potential.

In addition to chemistry, it is also worth noting that the type of cells used in these experiments differ between studies. Although, Cicco *et al*.^23^, and Lin *et al*.^37^, both use fibroblasts to test their cell response (and the same linkers), Cicco uses NHDF fibroblast, whereas Lin uses NIH/3T3, which may also contribute to the conflicting results. A study by Petushkov *et al*., used HEK-293 and RAW264.7 macrophages to assess silicalite nanoparticles functionalised with both thiol and amine linkers^27^. Petushkov’s study found that the effect of surface functionalisation on cytotoxicity was cell type dependent. For particles functionalised with Thiol, higher cytotoxicity was observed in RAW264.7 macrophages, which was attributed to the reduction in S-nitrosoglutathione and nitric oxide levels. However, once again direct comparison between studies is difficult, owing to the differences in silica particles tested. Nanoparticles are more likely to be directly phagocytosed whole by the cells, whereas particles of 20 *μ*m in diameter are more likely to be surrounded by macrophage cells which migrate to the surface of the particle to slowly break it down.

Inflammation is a normal healing process, however, prolonged inflammation due to non-cell-friendly biomaterials and/or by-products could result in an adverse inflammatory response that causes rejection by the host tissue^36^. Macrophage cells are the body’s first defence mechanism when any foreign material is implanted *in situ*. An understanding of the pro-inflammatory response can be obtained *in vitro* by measuring the level of interleukin-1(IL-1α) and tumour necrosis factor-alpha (TNF-α) secreted by J774.2 macrophages exposed to diatom frustules. These pro-inflammatory cytokines are measured using an enzyme-linked immunosorbent assay (ELISA) and the same experimental setup. IL-1α is considered a dominant IL1 cytokine in other pro-inflammatory murine model studies^38^, and it was therefore selected over IL1-β in this preliminary study. Lipopolysaccharide (LPS) derived from *Pseudomonas aeruginosa* was used as a positive control.

**Figure 6:**
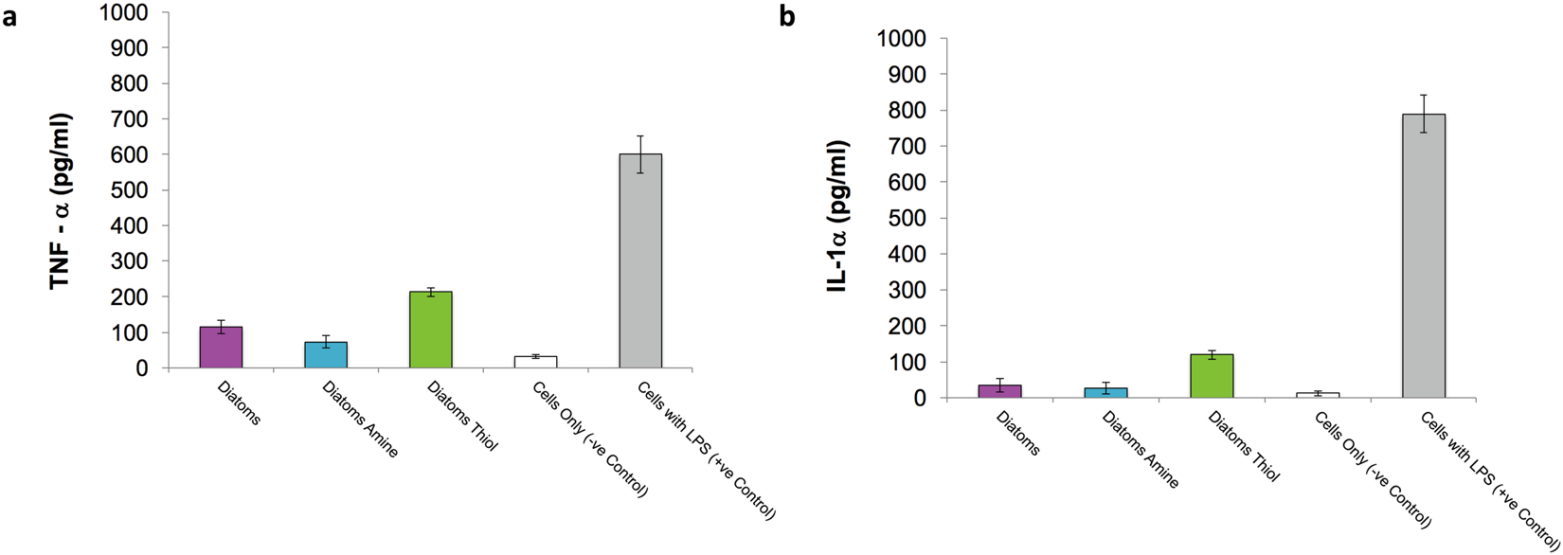
Pro-Inflammatory response of J774.2 macrophages grown on diatom frustules (**a**) TNF-α and (**b**) IL-1α release. Error barsindicate ±1 SD, N = 3.

IL-1α and TNF-α cytokines were released from J774.2 culture in the presence of diatom frustules (Fig. 6). Trace endotoxins in fetal calf serum (FCS) could potentially stimulate monocytes, a macrophage-cells only control was therefore tested. The release of IL-1α and TNF-α was highest in J774.2 cells exposed to Diatom Thiol, indicating that the thiol up-regulates a pro-inflammatory response. ANOVA indicated a treatment effect (p = 1.022 × 10^−^9) and post hoc testing confirmed that there was no significant difference between Cells Only and Diatom Amine treatment groups and both had a significantly lower inflammatory response than all other treatments. The Cells with LPS treatment had a significantly higher inflammatory response than all other treatments, followed by Diatoms Thiol, and Diatoms treatment groups. Similarly, results from the IL-1α assay identified a significant difference between groups (ANOVA p = 9.895 × 10^−14^). Post hoc test, Cells Only, Diatom, and Diatom Amine treatments showed no significant difference from one another and had a significantly lower inflammatory response than all other treatments. The Cells with LPS treatment had a significantly higher inflammatory response followed by Diatom Thiol treatment group.

**Figure 7:**
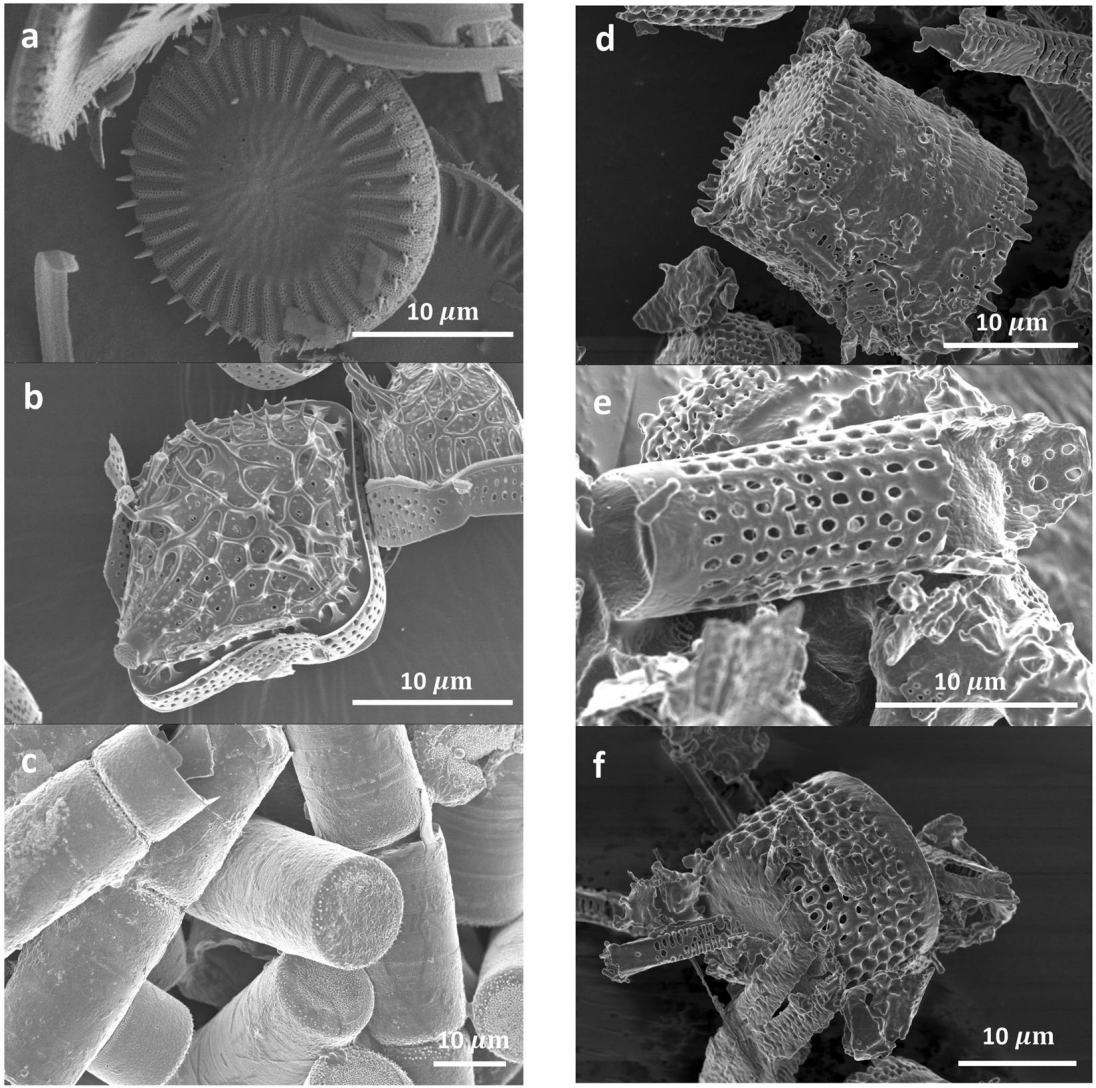
Scanning electron micrographs (**a**) C. meneghiniana (**b**) Triceratium dubium (**c**) Melosira varians (**d** to **f**) Diatomaceous earth (Note: this material is a crude mixtures of different species of fossilised diatoms, images represent image particles found in the mixture).

*C. meneghiniana* was chosen as a test species because of its availability but to compare the cell response to that from other species, cytotoxicity and cell proliferation was measured in response to two other types of diatoms, and to diatomaceous earth without functionalisation. In addition to *C. meneghiniana, Triceratium dubium* and *Melosira varians* were also tested. The morphology of these diatoms are shown in the SEM micrographs in Fig. 7. *T. dubium* (Fig. 7b) is a diamond shaped particle with sharp spines protruding from a network of ridges across the surface of the valve, whereas *M. varians* (Fig. 7c) are hollow tubular particles with a relatively smooth surface. Diatomaceous earth contains a crude mixture of different species of fossilised diatoms, Fig. 7d–f show some of the different structures of diatoms found in the sample tested in this study. For these cellular studies, both diatom frustules and conditioned media was tested with J774.2 cells as before, then the experiments were repeated with primary human bone marrow stromal cells (hBMSCs). Silica has been reported to induce a positive therapeutic response in bone healing, therefore, a preliminary experiment was conducted to test cytotoxic response of hBMSCs to different diatom frustules and assess their ability to proliferate when seeded on these types of substrates.

**Figure 8:**
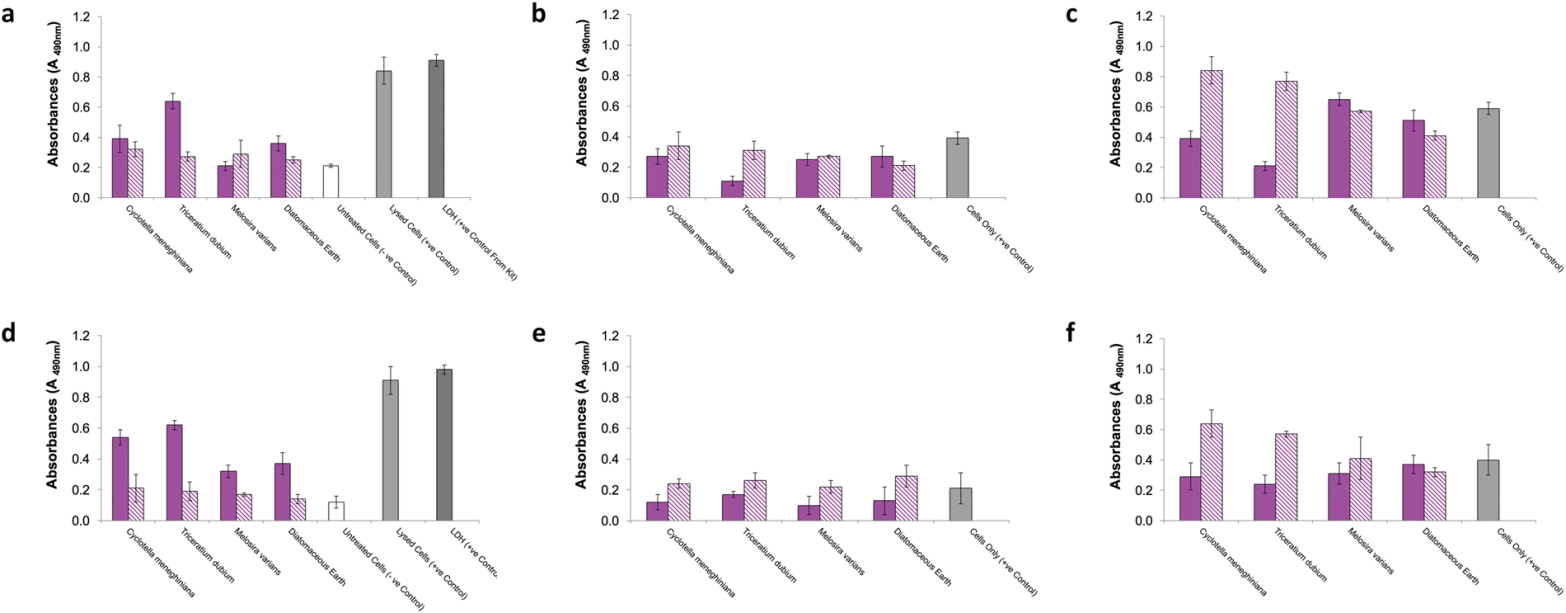
Biological Response of J774.2 macrophages and primary human bone marrow stromal cells (hBMSC) grown on diatoms listed in Fig. 7. (**a**) LDH release of J774.2 macrophages grown on diatom frustules (solid bar), conditioned media (lined bar) for 24 h. Cell proliferation of J774.2 macrophages grown on diatom frustules (solid bar), conditioned media (lined bar) for (**b**) 24 h (**c**) 72 h. (**d**) LDH release of hBMSC grown on (solid bar), conditioned media (lined bar) for 24 h. Cell proliferation of hBMSC grown on (solid bar), conditioned media (lined bar) for (**e**) 24 h (**f**) 72 h.

In all conditioned media groups, there was no difference in their cytotoxicity level compared to the negative control (untreated cells) in both cell types, indicating the conditioned media is non-toxic. Yet *T. dubium* frustules provoked the highest LDH release in both J774.2 cell (Fig. 8a) and hBMSC (Fig. 8d), perhaps indicating that its sharp spines were not well tolerated by the cells. Cell proliferation in *C. meneghiniana* and *T. dubium* followed the same trend in both cell types, with higher cell proliferation in the condition media groups (Fig. 8b, c, e and f). This effect became more pronounced after three days in both cell types. *M. varians* however, showed no difference in cell proliferation in J774.2 after one day (Fig. 8b) between diatom frustules and conditioned media groups, however, after three days (Fig. 8c), higher cell proliferation was observed in the diatom frustules group. The hBMSC however, followed the opposite trend, with the effect that higher cell proliferation was observed in *M. varians* conditioned media after one day (Fig. 8e), and no difference was observed after three days (Fig. 8f) between the diatom frustules and conditioned media groups. The conditioned media for diatomaceous earth, showed lower cell proliferation after both time points compared to the fossilised frustules in J774.2 cells. No difference was observed in cell proliferation at either time point in hBMSCs.

Few studies to date have tested the *in vitro* cytotoxicity of diatom frustules in the context of bone repair. Of the few studies, only Cicco *et al*.^23^, directly assesses the cell response of Saos-2, an osteoblast-like cell line on fresh diatoms (*T. weissflogii*). Cicco’s study reported higher cell proliferation in diatom frustules prior to functionalisation compared to diatoms functionalised with amine and thiol linkers, when tested with Saos-2 cells, which supports our findings (Fig. 5b) with macrophage cells. Lopez-A lvarez *et al*., study on comparing diatomaceous earth coatings to synthetic silica coatings, showed diatomaceous earth induced significantly better osteoblast proliferation with no cytotoxicity when tested *in vitro* using Saos-2 cells^39^. No other studies were found using bone cells.

The results for the different species of diatom studied shows that the surface nanotopography influences cell response, however, further studies are required to understand the underlying mechanisms that produce this effect. The results in Fig. 8 indicate that diatom frustules and their byproducts, which were tested in the form of conditioned media, could be considered as a non-cytotoxic biomaterial, and thus a suitable candidate for orthopedic repair. Diatom frustules offer a unique platform to create an index of cellular responses to material attributes, while releasing silicon ions in both implantable biomaterials and drug-delivery systems. The huge diversity of nanotopographies, shapes and sizes that naturally occur across the various species of diatom, and their ability to systematically reproduce identical structures, cannot be replicated synthetically. To put this in context, over 200,000 different diatom species exist with over 184 new species characterised on average per year^40^.

## Conclusion

The study has shown that diatom biosilica is non-toxic and does not invoke a pro-inflammatory response, thus making it a suitable material to profile the cellular response to different nanotopographies, enabling us to unravel the combined interfacial effects of nanotopography and silica dissolution. This model system will now be used to investigate the role of silica *in vivo*. Diatom biosilica offers a huge array of unique surface morphology, shapes and particle sizes which can be systematically mapped to help quantify the role of silica in bone repair and its applications in drug-delivery systems.

This study has shown that the interfacial effects of linkers, both amine and thiol, can have a significant effect on the cellular response *in vitro*. The thiol linker used in this study clearly induced a more detrimental effect on cellular activity than the amine linker. This work highlights the need for more systematic approaches in the selection of silica substrates used, in order to better understand the biological response to chemical linkers prior to their application in biomaterials or drug delivery systems.

## Methods

### Algal cell culture and harvest

*Cyclotella meneghiniana* was isolated into an axenic culture from Mississippi River (USA) collections obtained at a sampling location on St. Cloud State University’s campus. An individual *C. meneghiniana* cell was extracted from the Mississippi River sample for culture initiation using a borosilica micropipette. These cells were transferred in series through droplets of sterile WC media^41^ (See supplementary info) until the medium surrounding the cells was free of contaminant organisms, ensuring that the initial culture was unialgal. Isolation from this preliminary culture continued, until a bacteria-free stock culture was established. Approximately 80 L of stock culture was maintained in WC media, pH 7.0 within four 20 L glass containers at 12.5 °C on a 16:8 light/dark cycle at 200 µEm^−2^ s^−1^, achieving an approximate density of 50,000 cells/ml. Cell density was determined by direct enumeration of cultures via clove oil preparations^42^.

The 20 L stock cultures of *C. meneghiniana* were expanded up to a 35,000 L photobioreactor. Once the 20,000 cells/ml density was obtained, approximately half the photobioreactor volume was harvested (12,500 L) using an Origin Oil AA4 high-voltage pulse assisted aggregation system, achieving a cells paste consisting of ~20% solids. The wet harvested cell paste (≈100 kgs) was dried at 60 °C for 24 hours, yielding ≈ 20 kg of dried algal material. The organic biomass was removed using concentrated nitric acid (HNO_3_)^43^. The resultant diatom frustules were then washed and centrifuged repeatedly with deuterium-depleted water (DDW) until a pH of 7.0 to 7.4 was obtained. This process removed the organic material and residual nitric acid leaving behind the *C. meneghiniana’s* siliceous cell wall. The centrifuged biosiliceous material was dried at 60 °C for 24 hours, yielding ~ 1kg of dried *C. meneghiniana* biomass.

### Surface modification

The two linkers used in this study were 3-aminopropyltrimethoxysilane (APTMS) and 3-Mercaptopropylmethyl-dimethoxysilane (MPDMS), purchased from Gelest, Morrisville, PA, USA. Both silanes were stored under nitrogen prior to use. Prior to functionalisation diatom frustules were cleaned by soaking in ethanol and dH_2_O respectively, then oven dried for 1 hour at 110°C. Silanisation was achieved using a method similar to that described by Zhu *et al*.^44^. Briefly, after drying, the diatom frustules were closed in a flask and purged extensively with nitrogen. The reaction was carried out by refluxing 50 ml of anhydrous toluene at 70 °C for 2 h. 1 ml of APTMS (or MPDMS) was added dropwise through a syringe into diatom-toluene solution, once the toluene had reached a temperature of 70°C. The complete reaction was carried out under nitrogen. After the reaction, the toluene-linker solution was removed, then the diatom frustules were washed successively with fresh toluene, ethanol and dH_2_O before being dried in an oven at 110 °C for 1h. Diatom frustules were stored in a 15 ml sealed tube that was purged with nitrogen prior to use.

### Surface Characterisation

Diatom frustules were suspended in ethanol, sonicated and then the solution was pipetted onto carbon tape and left to dry. Once dry, samples were either gold coated for scanning electron microscopy (SEM), carbon coated for Energy dispersive X-ray spectroscopy (EDX analysis) or uncoated for X-ray photoelectron spectroscopy (XPS). SEM images were acquired on a Hitachi (SU8030) in secondary electron mode, with the electron beam operating at 2 kV and 10 μA. EDX data was acquired on a Hitachi (SU8030) with an Oxford AZtec X-max 80 SDD EDS detector. The compositional maps were carried out at 20 kV accelerating voltage. XPS was carried out on a Thermo Scientific ESCALAB 250Xi. Diatom frustules dried on carbon tape were mounted onto a stainless steel trough for XPS analysis. Survey scans and high-resolution scans were collected between, 0-1,100 eV binding energies respectively. Binding energies of spectra were referenced to the C 1s binding energy set at 284.8 eV. Six random points were collected per sample in triplicate for element composition evaluation. Prior to XPS measurements, the diatom-frustule powders were placed into the entry-loaded chamber to pump overnight. To verify the XPS results CHNS analysis to determine the bulk chemistry was performed on a Perkin Elmer PE2400CHNS.

### Diatom frustules preparation for cell culture

Diatom frustules were autoclaved at 121°C for 20 mins, then left to cool overnight. All experiments were carried out at concentrations of 50 mg/ml unless otherwise stated.

### J774.2 Cell Culture

The cell line J774.2 macrophages (ATCC, UK) were grown and maintained in RPMI 1640 medium (Invitrogen) supplemented with 10% fetal calf serum, FCS (Gibco), 100U/ml Pen/strep and 100mg/mL streptomycin (Invitrogen) at 37 °C in 5% CO_2_. For all cellular studies, a concentration of 50mg/ml of crude diatom biosilica (diatom frustules and griddle-bands) was suspended in media and incubated at 37 °C for 24 hours prior to plating with J774.2 macrophages. 50mg/ml was found to be the maximum concentration of crude diatom biosilica that could be homogenously suspended in media. J774.2 cells were seeded at a density of 1 × 10^4^ cells/cm^2^.

### Cytotoxicity Analysis

Lactic dehydrogenase release (LDH) was measured using a cytotoxicity assay kit (Cytotox®96 reagent Non-Radioactive kit, Promega Corp). Two positive controls were used for this assay, the LDH positive control from the kit and an aliquot of lysed cells, and one negative control of untreated cells. LDH is released when the cell membrane is damaged, therefore, this assay was used to assess the level of damage the diatom-biosilica caused to the J774.2 macrophages cells. The assay was performed as per manufacturer’s instructions. The absorbance signal was measured at 490nm using a Hybrid Multi-Mode Microplate Reader (BioTek, Winooski, VT). All readings measured were below 1. Live-dead staining and MTS were used to measure cell viability/proliferation using a LIVE/DEAD^TM^ Viability/Cytotoxicity Kit for mammalian cells (L3224, Invitrogen). For the Live/Dead assay, J774.2 macrophages cells were cultured on diatom frustules for 24 hours then stained with live/dead stain according to the manufacturer’s instructions. Briefly, cells were incubated for 5 to 10 minutes with 4μM calcein AM (live) and 4μM ethidium homodimer-1 (dead). Images were taken using a Leica fluorescence microscope equipped with a 485 ± 10nm optical filter for calcein AM (live cells) and a 530 ± 12.5 nm optical filter for ethidium homodimer-1 (dead cells). Images were processed using ImageJ (National Institute of Health, Bethesda, MD) with 6 live/dead images taken per well (total 6 images × 3 wells = 18 images per sample) to assess cell viability. Cell proliferation was carried out using a MTS assay (Promega Cell Titer 96 Aqueous One Solution Cell Proliferation Assay). After 24 h and 72 h of culturing J774.2 macrophages cells with diatom-biosilica, the MTS assay was performed as per manufacturer’s protocol.

### Cytokine Release

Interleukin-1 (IL- 1α) and tumour necrosis factor-alpha (TNF-α) was measured using an enzyme-linked immunosorbent assay (ELISA) (TNF-α: KMC3011, IL-1α: EMIL1A, both Invitrogen). Lipopolysaccharides (LPS) derived from *Pseudomonas aeruginosa* were added to tissue culture media at a concentration of 1 μg/mL as a positive control to upregulate TNF-α production and IL- 1α production by J774.2 macrophages.

### Silicon release profile from diatom frustules

*C. meneghiniana* diatom frustules were added to RPMI 1640 medium (Invitrogen) at a concentration of 50mg/ml in a 15 ml metal free centrifuge tube. Two samples were incubated, one for 24h and one for 72 h at 37°C, then centrifuged. The liquid fraction was filtered using a 2μm syringe filter (Gilson). The solid fraction was discarded. Aliquots of the conditioned media were used for ICP-OES analysis. Silicon concentration was determined using an Inductively coupled plasma mass spectrometry (ICP-OES) (Varian, VISTA-MPX Inc., CA, USA). A standard curve was prepared from 0.1, 0.5, 1, 2, 5 and 10 ppm Si standard (Inorganic Ventures) dissolved in 2% nitric acid to calibrate the instrument.

### Dose Response Conditioned Media preparation

*C. meneghiniana* diatom frustules were added to RPMI 1640 medium at concentrations of 75, 50 and 25mg/ml. A second sample of 25 mg/ml was prepared and diluted with RPMI 1640 medium without supplements to concentrations from 12.5 mg/ml to 0.195mg/ml. These samples were incubated for 24h at 37°C. After incubation, samples were vortexed. 2ml was taken from each sample and transferred into a fresh tube then centrifuged (the solid discarded) and filtered using a 2μm syringe filter ready for cell culture.

### Exploration of other diatom silica

*Triceratium dubium* and *Melosira varians* were grown using the same method previously described for *Cyclotella meneghiniana*. Diatomaceous earth was purchased from Sigma. All samples were autoclaved prior to cell culture. The concentration of diatom silica for this experiment was lowered to 25mg/ml owing to handling difficulties with some of the species. The LDH and MTS assays were performed as previously described. hBMSC cells were cultured in αMEM supplemented with 10% foetal bovine serum (Analab), 1% l-glutamine (Gibco, Invitrogen), and 1% Pen/strep solution (Gibco). Cells were passaged twice prior to use. For both LDH and MTS assays, cells were seeded at a density of 5 × 10^4^/cm^2^ at passage 3.

### Statistical Analysis

A one-way ANOVA comparison with a Tukey’s post hoc test was performed for all experiments evaluating cytotoxicity, cell viability, cell proliferation and cytokine release. Additionally, a t-Test was performed comparing 24 h and 72 h data for each individual treatment. For all statistical comparisons, a critical value of p* < *0.05 was used to test the null hypothesis, HO = all treatments responded identically. In each case, data did not violate assumptions of normality when tested and appeared not to violate other assumptions required for one-way ANOVA comparison. Analyses were conducted with IBM SPSS Statistic v.22, Armonk, NY. All error bars indicate ± 1 standard deviations (SD) for N = 3,unless otherwise stated in the legend of the figure or table.

## Electronic supplementary material

Supplementary Information

## References

[1] Hench LL (2006). The story of Bioglass. J. Mater. Sci. Mater. Med..

[2] Wilson J, Pigott GH, Schoen FJ, Hench LL (1981). Toxicology and biocompatibility of bioglasses. J. Biomed. Mater. Res..

[3] Bohner M (2010). Design of ceramic-based cements and putties for bone graft substitution. Eur. Cell. Mater..

[4] Jones, J. R. Review of bioactive glass: From Hench to hybrids. *Acta Biomaterialia* **9(1), **4457–4486 (2012). 10.1016/j.actbio.2012.08.023 22922331

[5] Anglin EJ, Cheng L, Freeman WR, Sailor MJ (2008). Porous silicon in drug delivery devices and materials. Advanced Drug Delivery Reviews.

[6] Mladenović Ž (2014). Soluble silica inhibits osteoclast formationan1. Mladenović, Ž. *et al*. Soluble silica inhibits osteoclast formation and bone resorption *in vitro*. Acta Biomater. 10, 406–18 (2014).d bone resorption *in vitro*. Acta Biomater..

[7] Bohner M (2009). Silicon-substituted calcium phosphates - a critical view. Biomaterials.

[8] Tan J, Saltzman WM (2004). Biomaterials with hierarchically defined micro- and nanoscale structure. Biomaterials.

[9] Place ES, Evans ND, Stevens MM (2009). Complexity in biomaterials for tissue engineering. Nat. Mater..

[10] Beck GR (2012). Bioactive silica-based nanoparticles stimulate bone-forming osteoblasts, suppress bone-resorbing osteoclasts, and enhance bone mineral density *in vivo*. Nanomedicine: Nanotechnology, Biology and Medicine.

[11] Yang L, Liu H, Lin Y (2015). Biomaterial nanotopography-mediated cell responses: experiment and modeling. Int. J. Smart Nano Mater..

[12] Christo S, Bachhuka A, Diener KR, Vasilev K, Hayball JD (2016). The contribution of inflammasome components on macrophage response to surface nanotopography and chemistry. Sci. Rep..

[13] Wang X, Schröder HC, Wiens M, Ushijima H, Müller WE (2012). Bio-silica and bio-polyphosphate: applications in biomedicine (bone formation). Current Opinion in Biotechnology.

[14] Wiens M (2010). The role of biosilica in the osteoprotegerin/RANKL ratio in human osteoblast-like cells. Biomaterials.

[15] Gordon R, Losic D, Tiffany MA, Nagy SS, Sterrenburg FAS (2009). The Glass Menagerie: diatoms for novel applications in nanotechnology. Trends in Biotechnology.

[16] Bromke M (2013). Amino Acid Biosynthesis Pathways in Diatoms. Metabolites.

[17] Sumper M, Brunner E (2006). Learning from diatoms: Nature’s tools for the production of nanostructured silica. Adv. Funct. Mater..

[18] Sumper M, Brunner E (2008). Silica biomineralisation in diatoms: The model organism Thalassiosira pseudonana. ChemBioChem.

[19] Tesson B (2009). Surface chemical composition of diatoms. ChemBioChem.

[20] Armbrust. E. V. The life of diatoms in the world’s oceans. *Nature* **459**, 185–192 (2009). 10.1038/nature08057 19444204

[21] Delalat, B. *et al*. Targeted drug delivery using genetically engineered diatom biosilica. *Nat. Commun*. **6**, 8791 (2015). 10.1038/ncomms9791 26556723

[22] Cicco SR (2016). Biosilica from Living Diatoms: Investigations on Biocompatibility of Bare and Chemically Modified Thalassiosira weissflogii Silica Shells. Bioengineering.

[23] Cicco SR (2016). Biosilica from Living Diatoms: Investigations on Biocompatibility of Bare and Chemically Modified Thalassiosira weissflogii Silica Shells. Bioengineering.

[24] Thrivikraman G, Basu B (2014). *In vitro*/*In vivo* assessment and mechanisms of toxicity of bioceramic materials and its wear. RSC Adv..

[25] Håkansson H, Chepurnov V (1999). A study of variation in valve morphology of the diatom *Cyclotella meneghiniana* in monoclonal cultures: Effect of auxospore formation and different salinity conditions. Diatom Res..

[26] Champion JA, Mitragotri S (2006). Role of target geometry in phagocytosis. Proc. Natl. Acad. Sci. USA..

[27] Petushkov, A., Intra, J., Graham, J. B., Larsen, S. C. & Salem, A. K. Effect of crystal size and surface functionalization on the cytotoxicity of silicalite-1 nanoparticles. *Chem. Res. Toxicol*. **22**, 1359–1368 (2009). 10.1021/tx900153k 19580308

[28] Santos J, Almeida SFP, Figueira E (2013). Cadmium chelation by frustulins: A novel metal tolerance mechanism in Nitzschia palea (Kützing) W. Smith. Ecotoxicology.

[29] Price NM, Morel FMM (1990). Cadmium and cobalt substitution for zinc in a marine diatom. Nature.

[30] Jaccard, T., Ariztegui, D. & Wilkinson, K. J. Incorporation of zinc into the frustule of the freshwater diatom Stephanodiscus hantzschii. *Chem. Geol.* **265**, 381–386 (2009).

[31] Friederichs RJ, Chappell HF, Shepherd DV, Best SM (2015). Synthesis, characterization and modelling of zinc and silicate co-substituted hydroxyapatite. J. R. Soc. Interface.

[32] Friederichs RJ, Chappell HF, Shepherd DV, Best SM (2015). Synthesis, characterization and modelling of zinc and silicate co-substituted hydroxyapatite. J. R. Soc. Interface.

[33] Bowler C, De Martino A, Falciatore A (2010). Diatom cell division in an environmental context. Curr. Opin. Plant Biol..

[34] Fowler, C. E., Buchber, C. & Delaco, C. An aqueous route to organically functionalized silica diatom skeletons. *Appl. Surf. Sci.* **253**, 5485–5493 (2007).

[35] Haines-Butterick LA, Salick DA, Pochan DJ, Schneider JP (2008). *In vitro* assessment of the pro-inflammatory potential of β-hairpin peptide hydrogels. Biomaterials.

[36] Haines-Butterick LA, Salick DA, Pochan DJ, Schneider JP (2008). *In vitro* assessment of the pro-inflammatory potential of β-hairpin peptide hydrogels. Biomaterials.

[37] Rabolli V (2014). The alarmin IL-1α is a master cytokine in acute lung inflammation induced by silica micro- and nanoparticles. Part. Fibre Toxicol..

[38] Rabolli V (2014). The alarmin IL-1α is a master cytokine in acute lung inflammation induced by silica micro- and nanoparticles. Part. Fibre Toxicol..

[39] López-Álvarez, M. *et al*. Silicon – hydroxyapatite bioactive coatings (Si – HA) from diatomaceous earth and silica. Study of adhesion and proliferation of osteoblast-like cells. *J Mat Sci: Mat Med*. **20**(5), 1131–1136 (2009). 10.1007/s10856-008-3658-0 19089599

[40] Julius *et al*. Pond Scum to Carbon Sink: Geological and Environmental Applications of the Diatoms 13, 1–13 (The Paleontological Society special publication, 2007).

[41] Guillard, R. R. L. In Culture of Marine Invertebrate Animals **1**, 29–60 (1975).

[42] McNabb, C. D. Enumeration of freshwater phytoplankton concentrated on the membrane filter. *Limnol. Oceanogr.* **5,** 57–61 (1960).

[43] Julius, M. L. & Theriot, E. C. The diatoms: a primer. Applications for the Environmental and Earth Sciences, Chapter 2, *Publishers: Cambridge University Press*, Editor John P Smol, Eugene F Stoermers, 8–22 (2010).

[44] Mojun Zhu, Maria Z. Lerum, and W. C. How to Prepare Reproducible, Homogeneous, and Hydrolytically Stable Aminosilane-derived Layers on Silica. *Langmuir* **28**, 416–423 (2012). 10.1021/la203638g PMC3243110 22128807

