# Supplementary material for: Exploratory Testing of Diatom Silica to Map the Role of Material Attributes on Cell Fate
Source: Sci Rep. 2017 Oct 26;7:14138. doi: 10.1038/s41598-017-13285-4 (PMC5658353; doi:10.1038/s41598-017-13285-4)
Supplement: Supplementary file 1 — Supplementary Information [file 41598_2017_13285_MOESM1_ESM.pdf]

Electronic Supplementary Information For

Exploratory Testing of Diatom Silica to Map the Role of Material Attributes on Cell Fate

Pamela J Walsh*[1,2], Susan A Clarke[3], Matthew Julius[4] & Phillip B Messersmith[2,5]

[1]School of Chemistry & Chemical Engineering, Queen's University, Belfast, UK,
[2]Biomedical Engineering Department, Northwestern University, Evanston, Illinois, USA,
[3]School of Nursing & Midwifery, Queen's University, Belfast, UK,
[4]Biological Sciences, St. Cloud State University, St. Cloiud, MN, USA.
[5]Departments of Bioengineering and Materials Science and Engineering, University of California, Berkeley, California, USA.

Corresponding Author: *pamela.walsh@qub.ac.uk

Address: School of Chemistry and Chemical Engineering, Queen's University Belfast, David Keir Building, Stranmillis Road, Belfast BT9 5AG, Northern Ireland, United Kingdom

Fax: +44 2890 97 5570

Tel.: +44 2890 97 4412

Supplementary Methods

Stock solutions of media were prepared and used throughout the culture phase of *C. meneghiniana, Triceratium dubium* and *Melosira varians.*

Major Nutrients	Quantity
1. $CaCl_2 \cdot 2H_2O$	36.76 mg/L
2. $MgSO_4 \cdot 7H_2O$	36.97 mg/L
3. $NaHCO_3$	12.60 mg/L
4. K_2HPO_4	8.71 mg/L
5. $NaNO_3$	85.01 mg/L
6. $Na_2SiO_3 \cdot 9H_2O$	28.42 mg/L
**Trace Elements**	
1. $Na_2 \cdot EDTA$	4.36 mg/L
2. $FeCl_3 \cdot 6H2O$	3.15 mg/L
3. $CuSO_4 \cdot 5H_2O$	0.001 mg/L
4. $ZnSO_4 \cdot 7H_2O$	0.022 mg/L
5. $CoCl_2 \cdot 6H_2O$	0.010 mg/L
6. $MnCl_2 \cdot 4H_2O$	0.180 mg/L
7. $Na_2MoO_4 \cdot 2H_2O$	0.006 mg/L
8. H_3BO_3	0.006 mg/L
**Vitamins**	
1. Thiamin·HCl	0.1 mg/L
2. Biotin	0.5 µg/L
3. Vitamin B_{12}	0.5 µg/L

Table S1. WC Media for algal culture - composition in deuterium-depleted water (DDW)

Bulk digest of Frustules: ICP-OES was used to characterise the elemental composition of the frustules. 0.1g of diatom frustules were completely dissolved in 10ml of 0.1 M solution of sodium hydroxide. The mixture was transferred into a round bottom flask and refluxed at 100°C for 48 hours under agitation. The mixture was then cooled and passed through a 0.22-micron filter to remove any solids remaining. The samples were tested for 22 elements (Al, As, Ba, Ca, Cd, Co, Cr, Cu, Fe, Mg, Mn, Ni, P, Pb, Sb, Se, Si, Sn, Sr, Ti, V, Zn). Any elements not reported in Table 1 were at concentrations below the detection limit of the instrument (<0.1ppm). The sample was tested by Queen's University Analytical Services facility.

SAMPLES	Al	As	Ca	Fe	Sn	Si	Zn
Diatom frustules	138 (±42)	3.7 (±1.2)	19929 (±297)	102 (±62)	356 (±249)	383178 (±932)	140.2 (±43)

Supplementary Table S2 (a): ICP-OES elemental profile of *C meneghiniana* frustules (bulk digest). Error bars indicate ±1 SD. N=3. Units = mg/kg

Verification of organic removal: Thermogravimetric analysis (TGA) was carried out on a SDT 2960 model from TA Instruments. All TGA analysis was performed under nitrogen flow with a heating rate of 20°C/min over a tempesrature range of 0 to 800°C.

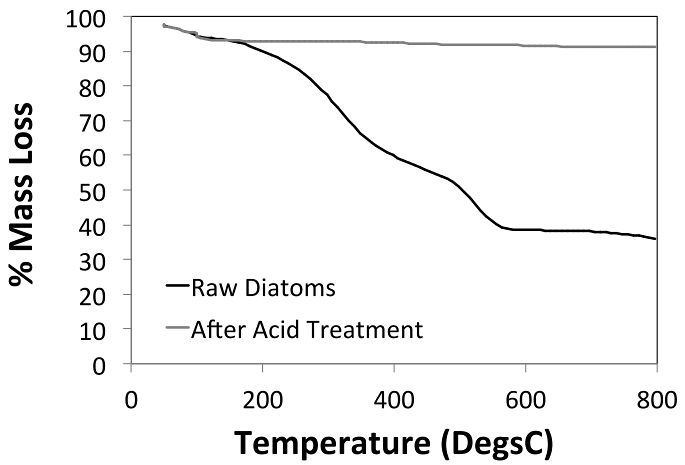

Supplementary Fig. S1: TGA mass loss profile of *C. meneghiniana* before and after nitric acid treatment.

The organic mass loss occurred between 200 to 600°C. After 600°C no further mass loss was observed, the mass plateaued, suggesting the remaining fraction was inorganic matter.

Specific Surface area and Pore Information: Gas absorption (BET) was used to determine the surface area and pore volume of *C meneghiniana* frustules. Nitrogen adsorption was carried out at 196°C using a Micrometrics Tristar II instrument at Queen's University Belfast. Samples were off-gassed overnight prior to analysis.

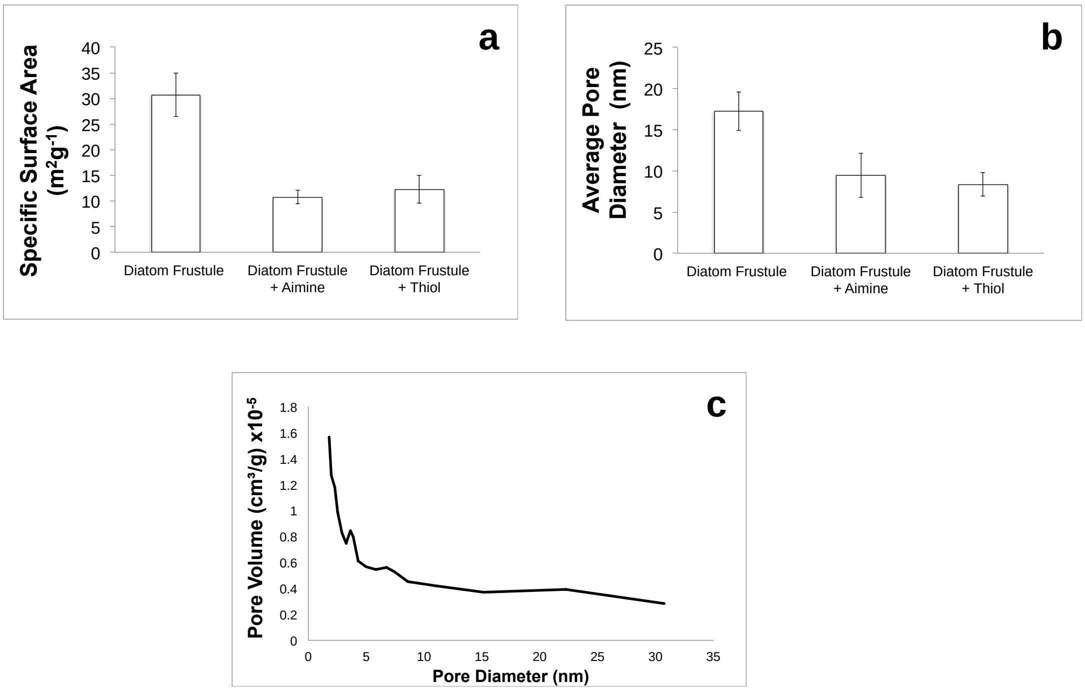

Supplementary Fig. S2: BET Analysis (a) Specific surface area (b) average pore diameter (BJH Adsorption) of diatom frustules with and without surface modification and (c) sample of pore volume versus pore diameter distribution for diatom frustules without modification. Error bars indicate ±1 SD. N=3.

Particle Size Analysis using SEM: *C meneghiniana* frustules were prepare for SEM using the same method described in the main manuscript. Top view images of the frustules were used to measure their average diameters. Two reads were taken one in the horizontal and one in the vertical direction on each frustule to ensure completely circular.

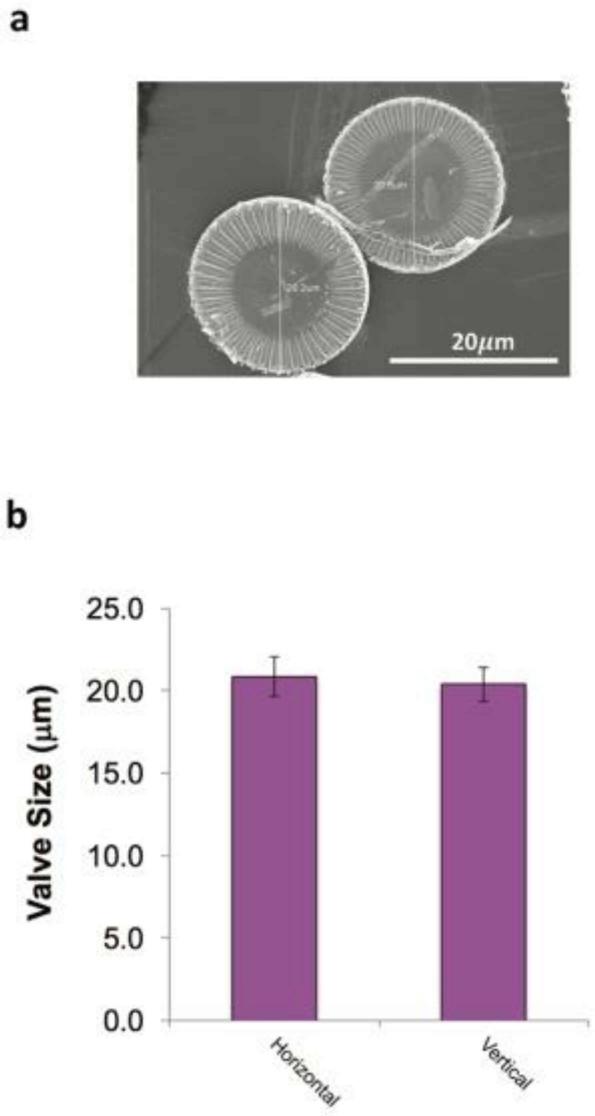

Supplementary Fig. S3: Scanning electron micrograph (a) of valves of *C meneghiniana* indicating point of measurement. (b) average valve size of *C meneghiniana* on horizontal and vertical direction. Error bars indicate ±1 SD. N=20.
